# Breast Cancer Germline Genetic Counseling and Testing for Populations of African Heritage Globally: A Scoping Review on Research, Practice, and Bioethical Considerations

**DOI:** 10.1200/GO.23.00154

**Published:** 2023-11-09

**Authors:** Yoshiko Iwai, Kadiata Toumbou, Takondwa Zuze, Jenny S. Morgan, Lusayo Simwinga, Sarah T. Wright, Yuri Fedoriw, Oluwadamilola T. Oladeru, Onyinye D. Balogun, Mya L. Roberson, Olufunmilayo I. Olopade, Tamiwe Tomoka, Shekinah N.C. Elmore

**Affiliations:** ^1^ University of North Carolina School of Medicine, Chapel Hill, NC; ^2^ UNC Gillings School of Public Health, Chapel Hill, NC; ^3^ UNC Project-Malawi, Lilongwe, Malawi; ^4^ Department of Medicine, University of North Carolina School of Medicine, Chapel Hill, NC; ^5^ Department of Clinical Medicine, Indiana University School of Medicine, Indianapolis, IN; ^6^ Department of Global Health, Indiana University School of Medicine, Indianapolis, IN; ^7^ UNC Health Sciences Library, University of North Carolina at Chapel Hill, Chapel Hill, NC; ^8^ Department of Pathology and Laboratory Medicine, University of North Carolina School of Medicine, Chapel Hill, NC; ^9^ Department of Radiation Oncology, University of Florida, Gainesville, FL; ^10^ Department of Radiation Oncology, Mayo Clinic in Florida, Jacksonville, FL; ^11^ Department of Radiation Oncology, Weill Cornell Medicine, New York, NY; ^12^ Department of Health Policy, University of North Carolina Gillings School of Global Public Health, Chapel Hill, NC; ^13^ Department of Medicine, University of Chicago, Chicago, IL; ^14^ Department of Radiation Oncology, University of North Carolina School of Medicine, Chapel Hill, NC

## Abstract

**PURPOSE:**

Despite the disproportionately high risk of breast cancer among women of African heritage, little is known about the facilitators and barriers to implementing germline genetic testing and counseling (GT/C).

**METHODS:**

This scoping review followed guidelines recommended by the Preferred Reporting Items for Systematic Reviews and Meta-Analyses extension for scoping reviews. Published manuscripts from database inception through 2021 were sourced from PubMed, Cumulative Index to Nursing and Allied Health Literature via EBSCO, Embase, Cochrane Library, and Scopus. Search terms were used to retrieve articles addressing (1) African heritage, (2) breast cancer, and (3) GT or GC. The screening involved abstract and title review and full-text review. Data were extracted for all articles meeting the inclusion criteria.

**RESULTS:**

A total of 154 studies were included. Most studies that took place were conducted in the United States (71.4%), and most first authors (76.9%) were from the United States. GT was conducted in 73 (49.7%) studies. *BRCA1*/*BRCA2* were the most commonly studied genes for germline mutations. GC was conducted in 49 studies (33.3%), and perspectives on GC were evaluated in 43 (29.3%). The use of racial/ethnic categories varied broadly, although African American was most common (40.1%). Racism was mentioned in three studies (2.0%).

**CONCLUSION:**

There is a growing body of literature on GT/C for breast cancer in women of African heritage. Future studies on GT/C of African populations should consider increased clarity around racial/ethnic categorizations, continued community engagement, and intentional processes for informed consent.

## Teaser

A scoping review on germline genetic testing for breast cancer among women of African heritage identifies a growing body of literature and key bioethical considerations for genetic testing/counseling.

## INTRODUCTION

Breast cancer mortality rates in the United States have decreased since 1989.^1^ However, these reductions in mortality are not appreciated across patient groups equally. Black women have death rates that are 40% higher than White women and have the lowest 5-year survival of any racial/ethnic group across tumor subtype, hormone receptor status, and disease stage.^1^

Poor outcomes in patients who are racialized as Black are seen across geographies. Breast cancer is the second leading cancer among women of African heritage,^2^ and breast cancer–associated mortality ranks the highest in the world, with a disproportionately low 5-year survival of 66% in contrast to high-income countries where survival approaches 90%.^3,4^ Country-specific survival estimates are as low as 12% in Uganda and neighboring countries.^5^

One area of research that seeks to explain these disparities is through the lens of genetics. Genetic testing (GT) is part of standard of care, but uptake has been relatively low.^6^ Some studies have reported genetic differences among racial/ethnic groups that may be partially attributable to population-level disparities. For example, women of African heritage are more likely to present with triple-negative breast tumors (TNBC), which are less responsive to standard treatment.^7,8^ Geographic subsets of TNBC prevalence have been reported at higher frequencies in West African regions than in African American or White American populations.^9^ Yet, studies have also noted that patients of African heritage are often not included in genetic studies.^10-13^

Globally, there is limited literature on genetic testing and counseling (GT/C) that considers how racial groups are constructed in different time periods and geographies. Furthermore, racism and its impacts on inclusion and access to GT/C at a global scale are underexplored. GT/C has been used as a standard model in high-income countries,^14^ and is suggested to increase cancer-related knowledge, perceived personal control, and risk perception accuracy, and decrease cancer-related anxiety and decisional conflict among patients.^15^ Further, GT can affect surgical planning and play an essential role in the treatment course.^16-18^ In TNBC, deleterious mutations have been reported at higher rates, and GT/C for *BRCA1* and *BRCA2* has been recommended regardless of age or family history.^19^

Despite the disproportionate disease burden and potential benefits for improving screening and treatment, GT/C does not appear to be widely incorporated into breast cancer care for women of African heritage. GT must be delivered with rigorous consideration of sociocultural, economic, and ethical contexts,^20-22^ and guidelines for GT/C implementation in populations of African heritage have yet to be standardized. The purpose of this scoping review^23^ was to evaluate the literature on germline GT/C for breast cancer in women of African heritage. We aimed to assess previous germline breast cancer GT/C applications, identify barriers and facilitators for implementation, and identify gaps in the present literature.

## METHODS

This scoping review was conducted with the guidelines recommended by the Preferred Reporting Items for Systematic Reviews and Meta-Analyses extension for a scoping review.^24^

A trained clinical health sciences librarian (S.T.W.) performed our comprehensive electronic search of publications using the following databases: PubMed, Cumulative Index to Nursing and Allied Health Literature via EBSCO, Embase, Cochrane Library, and Scopus. Our search was not restricted by language. All results were collected from the database's inception through July 4, 2021. Search terms were used to retrieve articles addressing the three main concepts of the search strategy: (1) African heritage, (2) breast cancer, and (3) GT or genetic counseling (GC; Appendix 1). The search strategy was conducted in PubMed using keyword and MeSH combinations. Other database search strategies included text word searching and database-specific thesaurus terms, if available. Results were downloaded to EndNote, and duplicates were removed. All references were uploaded to Covidence Systematic Review software (Covidence, Melbourne, Australia), a web-based tool designed to track each step of the abstraction and review process.

To evaluate germline GT/C in women of African descent, the decision was made to broadly include racial/ethnic categories that could consist of individuals of African heritage and relate to the overlapping but distinct exposures of inheritance and global anti-Black racism. Studies with African American, Black, or Black American participants were included. Of note, the term sub-Saharan Africa was used throughout the search process. Still, the authors have moved away from this terminology for a more accurate and equitable representation of the study focus.^25,26^ The authors also use the term heritage to acknowledge the broad scope of genetic, sociocultural, and historical contexts that are passed down to individuals and holistically capture how communities may choose to define themselves. The term ancestry was generally avoided as identifying biological correlates of race was not the primary goal of this study.

Inclusion criteria were studies published before July 4, 2021; involving GT/C of human participants for breast cancer; including participants of African heritage by ancestry informative markers or self-report; and quantitative, qualitative, or mixed-methods studies. Exclusion criteria were incorrect study type, focus, intervention, population, cancer type, or article type (eg, commentaries). All articles were screened by title and abstract by two reviewers (Y.I., K.T., J.S.M., or S.N.C.E.). A third reviewer resolved conflicts (J.S.M. or S.N.C.E.). Selected articles underwent full-text review, where inclusion was determined based on the manuscript's contents. Two independent reviewers conducted this step (Y.I., K.T., J.S.M., or S.N.C.E.), with a third reviewer for conflict resolution (J.S.M. or S.N.C.E.).

Data extraction was conducted in an online form (Appendix 2) developed by the research team (Qualtrics XM, Provo, UT). A single reviewer extracted each article, and a second reviewer was consulted as needed. After data extraction, thematic content analysis was conducted. One reviewer (Y.I.) completed a line-by-line analysis and assigned preliminary themes. These themes were assessed by a second reviewer (S.N.C.E.), and conflicts were resolved through discussion.

Reflexivity was addressed throughout the analysis by including a diverse research team.^27^ The authors involved were cognizant of their own experiences with GT/C, racism, and cancer care. The team was composed of individuals who self-identified as Black/African American, Asian, and White.

### Ethics Approval Statement

This is a literature review and did not require ethics approval.

## RESULTS

### Study Location and Authorship

**FIG 1:**
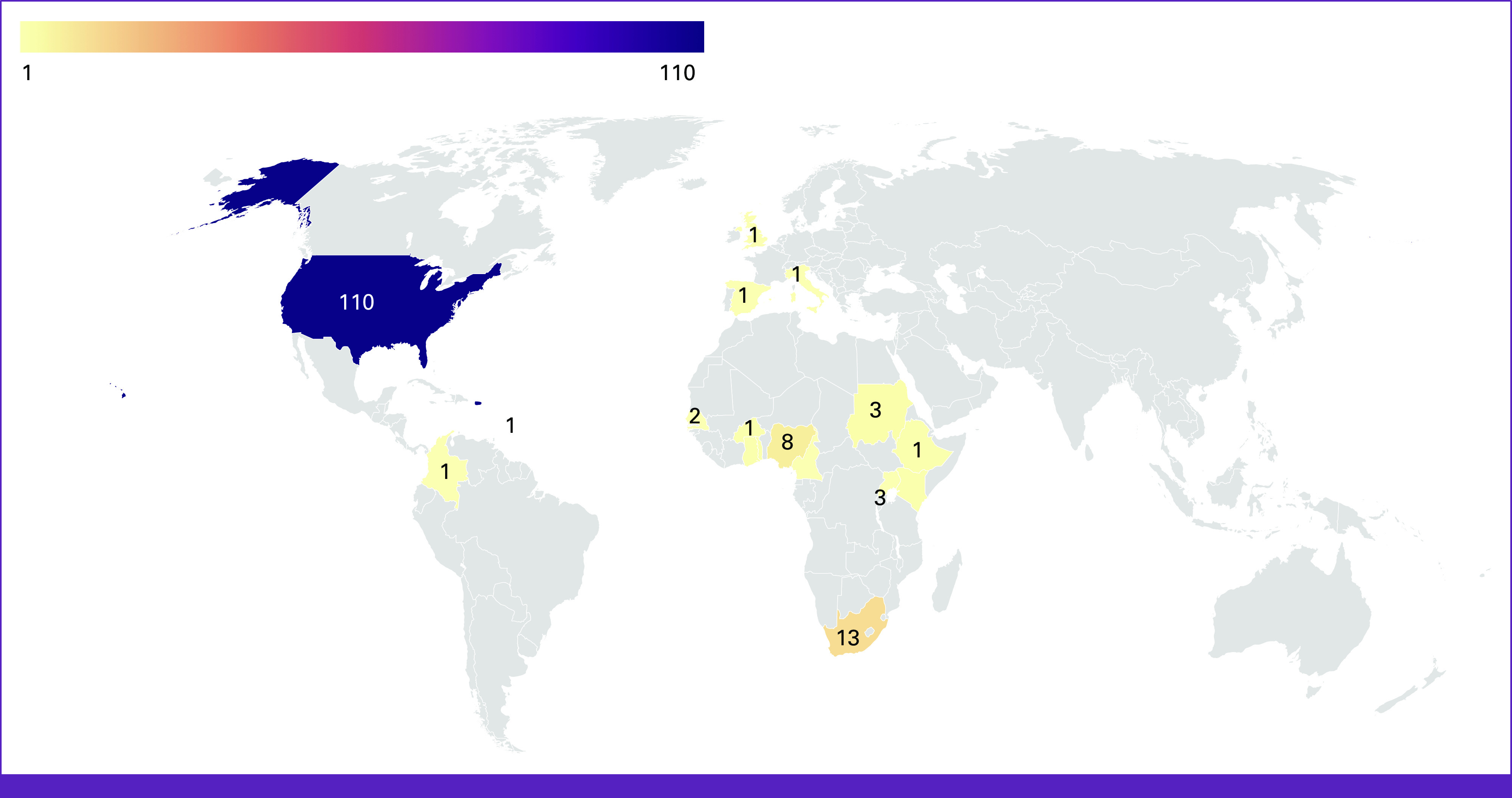
Map of countries included in scoping review. Geographic representation of studies on germline genetic testing/counseling for breast cancer.

The initial search yielded 2,890 articles. The distribution of articles per database is summarized in Figure 1. There were 1,820 articles remaining after duplicates were removed. After title and abstract screening, 503 articles were eligible for full-text review. A total of 154 studies met the inclusion criteria and were ultimately included.

**FIG 2:**
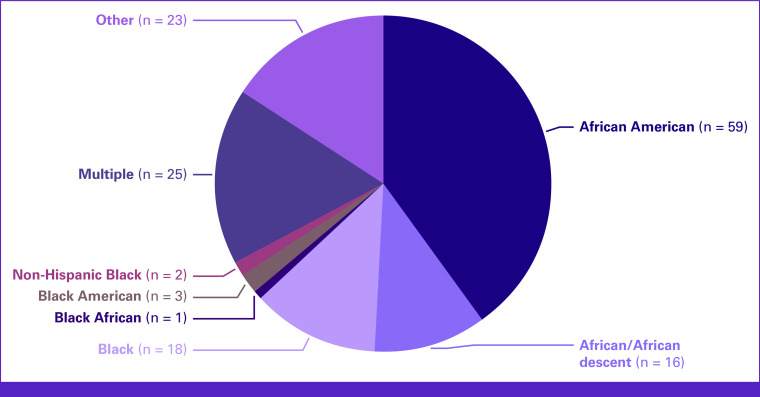
Pie chart of racial categories used by included studies.

The countries where the studies took place are represented in Figure 2. Most studies were conducted in the United States (71.4%), followed by South Africa (8.4%), Nigeria (5.2%), Rwanda (1.9%), Sudan (1.9%), Ghana (1.3%), Kenya (1.3%), Senegal (1.3%), and Togo (1.3%). Five studies were conducted in multiple countries. The geographic spread of the first and last authors is summarized in Table 1, with most first authors (76.9%) and last authors (78.2%) being from the United States

**TABLE 1:**
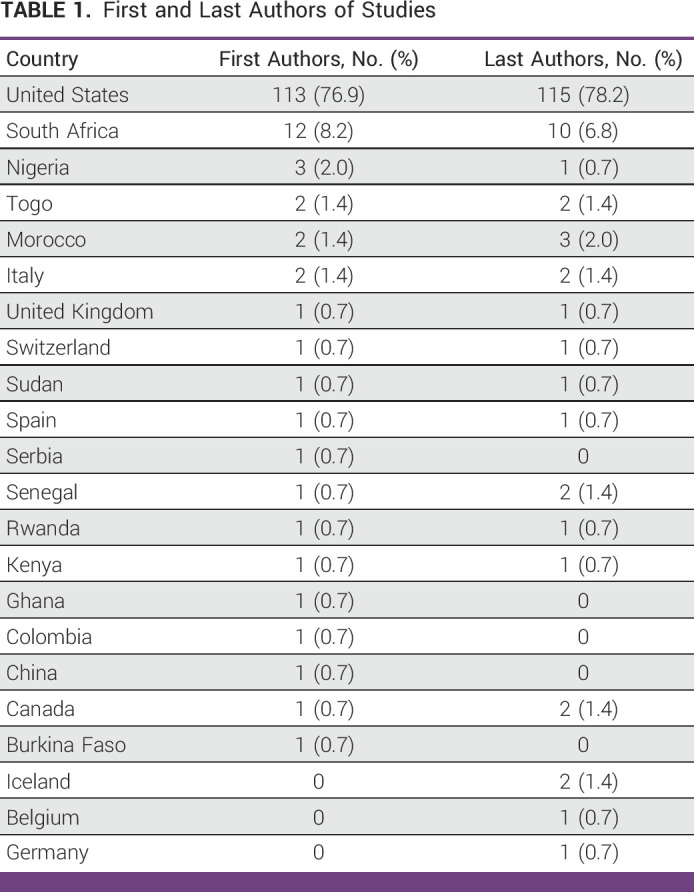
First and Last Authors of Studies

### Study Design, Data Sources, and Informed Consent

The characteristics of the studies that were included are summarized in Table 2. Most studies (65.3%) were prospective, including cohort and cross-sectional studies. Total population sizes varied, with approximately half of the studies (45.6%) having 100-500 participants. There were 41 studies (27.9%) that used an existing database, including state cancer registries or the SEER Program. Informed consent was obtained in 73.8% of studies and not obtained in 11.0%. There were 22 (15.3%) studies where informed consent status was unclear.

**TABLE 2:**
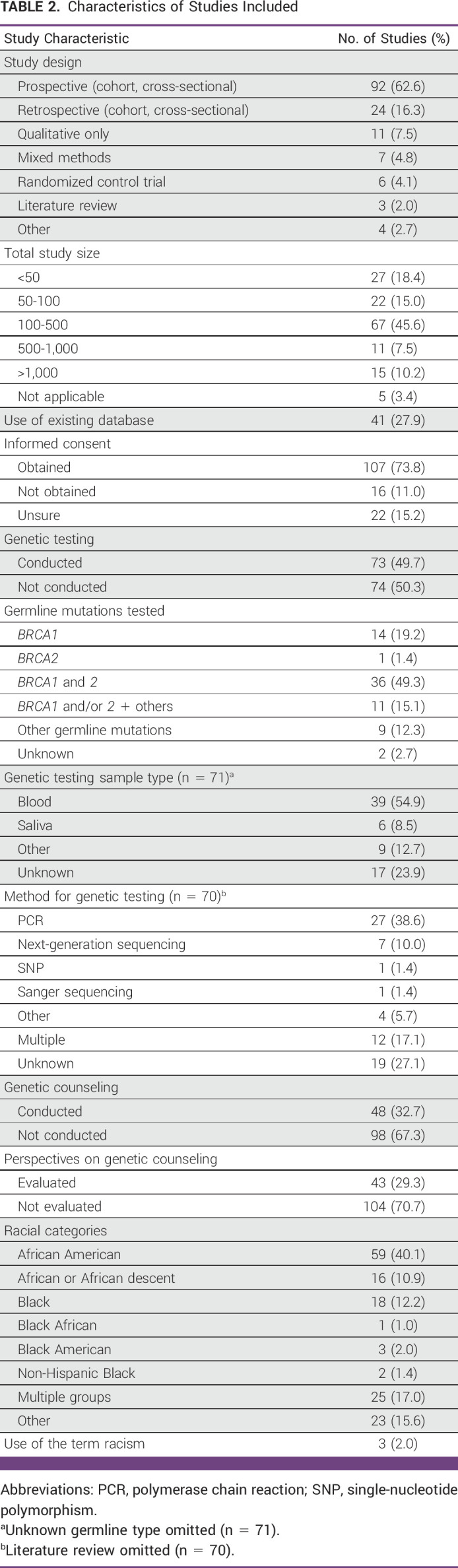
Characteristics of Studies Included

### GT: Target Genes, Methods, and Variants of Uncertain Significance

Seventy-three (49.7%) studies conducted GT (Table 2). *BRCA1* and *BRCA2* were tested in 49.3% of studies, followed by studies that only tested *BRCA1* mutations (19.2%) and for *BRCA1* and/or *BRCA2* mutations with other germline mutations (15.1%). Non-*BRCA* germline mutations were assessed in 12.3% of studies, including *TP53*,^28-30^ *ATM*,^31^ *CHEK2*,^32^ *PALB2*,^33-35^ and *DARC/ACKR1*.^9^ GT was done using peripheral blood most commonly (54.9%) and saliva (8.5%). GT was conducted using polymerase chain reaction (38.6%), next-generation sequencing (10.0%), single-nucleotide polymorphism (1.4%), and Sanger sequencing (1.4%). Many studies reported on variants of uncertain significance (VUS).^28,30,33-61^

Most studies (60.3%) that reported on GT were conducted in the United States. Ten (13.7%) GT studies were conducted in South Africa, three (4.1%) were conducted in Nigeria and Rwanda, and less than two studies were conducted in other non-US countries including Sudan and Cameroon. Non-US studies had relatively smaller sample sizes (studies with <100 subjects seen in 48.3% of non-US studies *v* 31.2% of US studies). Three non-US studies focused on participants at high risk of breast cancer (eg, patients with Li Fraumeni syndrome).^29,45,58^ Non-US studies varied in their recommendations for GT, with several recommending GT for breast cancer screening because of higher rates and/or risk of germline mutations,^40,45,56,62,63^ and others explicitly arguing against GT because of lower prevalence and/or risk of germline mutations related to breast cancer.^28,30,32,64,65^

### Genetic Counseling

GC was conducted in 48 studies (32.7%), and perspectives on GC were evaluated in 43 (29.3%; Table 2). The majority (95.8%) of these studies were conducted in the United States, with two non-US studies from Kenya and South Africa.^41,66^ Of the studies that assessed perspectives on GC, 37.2% used surveys, 28.0% used individual interviews, 18.6% used multiple assessment methods, and 9.3% used focus groups.

Three studies assessed factors associated with the likelihood of receiving GT/C,^67-69^ and one explored eligibility for GT/C.^70^ Several studies reported rates of GT/C were lower among Black/African American women compared with White women, with rates of GT/C as low as 16 times less likely in Black women.^42,44,45,71^ Notably, three studies reported no differences in GT/C receipt by race.^72-74^ Several studies evaluated acceptance or satisfaction of *BRCA* testing; GT/C participation and recruitment was generally high (60%-80%) with rates >80% for participant satisfaction with their decision to undergo GT/C.^75-79^ Multiple studies explored other elements of GT/C, such as negative emotional reactions and rates of depression.^74,80-84^ Study findings varied from no baseline differences in GT/C-related distress to higher rates among women of African heritage.

Six studies implemented strategies for improving GT/C rates, including culturally competent genetic counselors and community-based GC practices.^85-90^ Knowledge around GT/C was assessed,^68,76,91-93^ with some studies reporting lower GT/C knowledge among Black or African American participants compared with White participants.^68,91^ Sociocultural beliefs were explored by multiple research groups, including medical mistrust.^81,94-96^

### Definitions of Race, Ethnicity, and Racism

The use of racial/ethnic categories varied. The breakdown of the racial categories is visualized in Figure 2. The most common racial category was African American (40.1%). Other categories included African or African descent (10.9%), Black (12.2%), and Black American (2.0%). Multiple groups were used to describe individuals of African ancestry in 17.0% of studies, and 15.6% used other racial/ethnic groups (eg, Black African, Black British, and Black Caribbean^97^). Racial/ethnic categories were clearly defined in 15 studies (10.2%). For example, Friebel et al^98^ indicated participants were inferred to have African ancestry based on self-report or inference based on place of birth or residence. Some studies included single racial/ethnic groups but clearly stated these categories, for example, “Our sample consisted of 63 individuals from a single African American (Creole) kindred.”^99^ Approximately 75% of studies had no definition of race/ethnicity in their research methods.

The word “racism” was included in three studies (2.0%) conducted in 2006, 2008, and 2020.^76,100,101^ No other studies included the term racism in any part of their manuscript. Kinney et al^76^ assessed perspectives of racism in general and in the health care setting using a validated Perceptions of Prejudice scale. Edwards et al^100^ included the potential role of racism in introducing and discussing their study on attitudes toward *BRCA1/2* testing among women of African heritage. Peterson et al^101^ contextualized their findings of hereditary breast cancer assessment disparities by discussing institutional racism and the historical context of the US Public Health Service Syphilis Study at Tuskegee.

## DISCUSSION

In this study, we sought to explore germline GT/C for breast cancer in women of African heritage. Studies were heterogeneous in size, geographic spread, study goals, and findings. Key themes from this scoping review may serve further research and help shape how we consider the intersections of race, heritage, genetics, and the overarching frame of systemic racism in germline GT/C.

Although disparities in outcomes or access were a focus of many studies, reported findings ranged from no racial/ethnic differences^74^ in GT receipt to up to 16-fold differences between populations of African heritage versus White populations.^42,44,45^ Similarly, GT/C recommendations ranged from testing according to patient autonomy (even against physician recommendations)^102^ to GT in all young Black women.^103^ GT/C disparities varied across studies, likely because of differences in study size, location, sociocultural differences in African or African American subgroups, and differences in chosen comparators. Populations of African heritage were the primary focus of this study, but most studies were physically conducted in the United States

We also identified a high volume of studies that reported VUS among populations of African heritage. As suggested by some studies, these findings are likely a result of less-accessible GT data in specific populations.^104^ This may be due to limited GT/C access and the sociocultural and historical contexts of GT/C rather than lack of interest as demonstrated by the high rates of GT/C acceptance, adherence, and satisfaction reported among African and African American populations.^75-79^

Few studies (25%) provided definitions of race/ethnicity, and there was broad heterogeneity in the categories used to describe groups. These findings are consistent with bioethics scholarship, which report unclear racial/ethnic categorizations across genetics literature and concern for insufficient rigor because of inconsistencies.^105-107^

Furthermore, US studies often compared White versus African American populations without clear rationale for their limited comparison groups.^42,52,74,91,101,102,108^ Non-US studies were under-represented in this review; however, some non-US studies included broader racial categories.^9,55^ Given the general under-representation of individuals of African heritage in genetic and cancer registries, the genes that are tested in these studies may not be capturing the appropriate genes for the specific populations of interest.^109^ More robust inclusion of individuals of African heritage is essential for understanding which genes to test and develop study designs with purposeful comparisons.

Within genetics literature, some researchers argue for the continued use of historic racial/ethnic categories because they are thought to provide crude but meaningful information about disease states.^11,12,106^ Others have embraced race as a social construct, given the greater genetic diversity observed within groups than between them and the mutability of racial categorizations across time and place.^105,110,111^ Some genetics research has transitioned from typological categories to terms like ancestral group and geographic population. Still, the arbitrary categories continue to risk perpetuating racist typological notions.^112^

Empirical studies on racial/ethnic categories and their impact on clinical outcomes are sparse. Yet, small-scale analyses of genetics researchers reveal an absence of systematic mechanisms for racial grouping and instead reliance on typological beliefs that are scientifically unexamined implying tenuous scientific merit.^105^ Despite including contemporary studies, our findings show ongoing inconsistencies in racial groupings. Determining which categories were most clinically valuable was outside this study's scope; however, our findings underscore the importance of clearly defining racial/ethnic categories and documenting the intent for which they are meant to serve.

Genetics research is fraught with historic and present racism, yet only three studies explicitly discussed racism.^11,113^ Racial disparities were a common focus, and the dearth of explicit mentioning of, or grappling with, the context in which GT/C research takes place is another concern for ethicality and intent behind large-scale germline testing. Further reckoning with racism is warranted to develop a meaningfully race-conscious approach to GT/C.

Another theme that emerged was the investigator's relation to the local community. There were several studies where first or last author affiliations differed from the patient population or the study's geographic region. This is consistent with studies that report high rates of genetics research about African-descendant populations coming from Europe and the United States and the risks of authorship parasitism.^114-116^ Community engagement is a critical investment for conducting ethical research and improving patient outcomes.^117,118^ Involvement of the local community and key stakeholders is even more pressing when the research focuses on ethnic minorities or a global context where resource accessibility varies by geography.^119,120^

For studies where the investigator's affiliation differed from the country where the research was conducted, it was often unclear how the local community was involved; this was also true for studies conducted within the United States. These discrepancies may be partially addressed by standardized inclusion of the local community by institutional review boards and upholding rigorous publication criteria by academic outlets.^27^

Despite the high volume of research conducted outside the researcher's institution, the role of informed consent was difficult to discern in nearly 15% of studies. Such studies would benefit from clarifying consent processes, and publication outlets may benefit from ensuring sufficient verbiage to uphold appropriate ethical standards.

Contrary to our expectations, few studies discussed the role of data ownership in GT/C research. Disclosure of personal data regarding GT/C results was explored by one study,^121^ but larger-scale studies on data privacy, storage, and usage were sparse. Further studies are warranted to understand the ethics of GT/C data ownership, security, privacy, and patients' perspectives regarding these issues.

The scoping review was selected as a review strategy because of the unknown quantity and quality of literature on GT/C in women of African heritage.^23^ Quality assessment was not conducted because of heterogeneity of article types. This study was primarily conducted at a US academic center. Although the focus was on individuals of African heritage and members of the team were affiliated with a hospital in Malawi, the review methodology may be biased toward high-income countries. We attempted to address this by including all languages; however, no non-English studies were ultimately included.

In conclusion, in this scoping review of GT/C for breast cancer in women of African heritage, we identified a growing body of literature that was conducted predominantly in the United States by researchers in the United States Approximately half of the studies carried out GT, most commonly of *BRCA1* and *BRCA2,* while one third conducted GC. Racial categories varied broadly, with insufficient definitions in most studies. Future research on GT/C for African heritage populations should consider increased clarity around racial/ethnic categorizations, more robust local community engagement, and intentional processes for informed consent and data ownership.

## Data Availability

All data can be made available upon reasonable request.

## APPENDIX 1. DATABASE SEARCH STRATEGIES

### PubMed

Searched February 7, 2021

1,056 results

("African descent" OR "African ancestry" OR "African Europeans*" OR "African Asians*" OR "African Americans*" OR "African migrants" OR "Southern African Development Community" OR "SADC" OR "African Caribbeans*" OR african continental ancestry group[mesh] OR “African continental ancestry” OR "Africa South of the Sahara"[Mesh] OR sub-sahara* OR subsahara* OR Angola OR Luanda OR Luanda OR Benin OR Porto-Novo OR Botswana OR Gaborone OR Burkina Faso OR Ouagadougou OR Burundi OR Gitega OR Cameroon OR Yaounde OR "Central African Republic" OR Bangui OR Chad OR N'Djamena OR Comoros OR Moroni OR "Democratic Republic of the Congo" OR Kinshasa OR "Republic of the Congo" OR Brazzaville OR "Cote d'Ivoire" OR Yamoussoukro OR Djibouti OR "Equatorial Guinea" OR Malabo OR Eritrea OR Asmara OR Eswatini OR Mbabane OR Ethiopia OR "Addis Ababa" OR Gabon OR Libreville OR Gambia OR Banjul OR Ghana OR Accra OR Guinea OR Conakry OR Guinea-Bissau OR Bissau OR Kenya OR Nairobi OR Lesotho OR Maseru OR Liberia OR Monrovia OR Madagascar OR Antananarivo OR Malawi OR Lilongwe OR Mali OR Bamako OR Mauritania OR Nouakchott OR Mauritius OR Port Louis OR Mozambique OR Maputo OR Namibia OR Windhoek OR Niger OR Niamey OR Nigeria OR Abuja OR Rwanda OR Kigali OR "Sao Tome and Principe" OR "Sao Tome" OR Senegal OR Dakar OR Seychelles OR "Sierra Leone" OR Freetown OR Somalia OR Mogadishu OR "South Africa" OR Pretoria OR South Sudan OR Juba OR Sudan OR Khartoum OR Tanzania OR Dodoma OR Togo OR Lomé OR Uganda OR Kampala OR Zambia OR Lusaka OR Zimbabwe OR Harare OR "west-africa*" OR "west africa*" OR "south african*" OR "east-africa*" OR "east africa*") AND (Breast Neoplasms[mesh] OR “breast neoplasm*” OR “breast cancer*” OR “breast neoplasm*” OR “cancer of the breast” OR “neoplasm of the breast” OR “neoplasms of the breast” OR “ductal breast carcinoma*” OR “mammary cancer*” OR “mammary neoplasm*” OR “breast malignant neoplasm*” OR “breast malignant tumor*” OR “breast malignant tumour*” OR “breast tumor*” OR “breast tumour*”) AND (((germline AND (breast[tiab] OR brca[tiab])) OR breast neoplasms/genetics[mesh] OR brca*[tiab] OR brca1 protein/genetics[mesh] OR brca2 protein/genetics[mesh] OR genes, brca1[mesh] OR genes, brca2[mesh]) OR brca1 protein, human[mesh] OR brca2 protein, human[mesh] OR Carcinoma, Ductal, Breast / genetics* OR Germ-Line Mutation[mesh] OR “germline mutation*” OR “germ-line mutation*” OR genes, p53[mesh] OR “p53 gene*” OR "TP53TG1 protein, human" [Supplementary Concept] OR "Tumor Suppressor Protein p53"[Mesh] OR "TP53 protein, human" [Supplementary Concept] OR "TP53TG5 protein, human" [Supplementary Concept] OR tp53[tiab] OR tp53tg1[tiab] OR tp53tg5[tiab] OR “TP53TG1 protein, human" [Supplementary Concept] OR "Tumor Suppressor Protein p53"[Mesh] OR "TP53 protein, human" [Supplementary Concept] OR "TP53TG5 protein, human" [Supplementary Concept] OR "TP53TG1 protein, human" [Supplementary Concept] OR "Tumor Suppressor Protein p53"[Mesh] OR "TP53 protein, human" [Supplementary Concept] OR "TP53TG5 protein, human" [Supplementary Concept] OR Tumor Suppressor p53-Binding Protein 1[mesh] OR "Rad52 DNA Repair and Recombination Protein"[Mesh] OR rad52[tiab] OR "Mutation"[Mesh] OR "Sequence Analysis, DNA"[Mesh] OR “dna sequence analysis” OR "Nanopore Sequencing"[Mesh] OR “nanopore sequenc*” OR

"High-Throughput Nucleotide Sequencing"[Mesh] OR “nucleotide sequenc*” OR "Sequence Analysis, DNA"[Mesh] OR “sequence analysis” OR "Nanopore Sequencing"[Mesh] OR “nanopore sequenc*” OR "High-Throughput Nucleotide Sequencing"[Mesh] OR “Nucleotide sequen*” OR "DNA Repair-Deficiency Disorders"[Mesh] OR “dna repair” OR "Li-Fraumeni Syndrome"[Mesh] OR “li-fraumeni” OR DNA, Neoplasm / genetics[mesh] OR DNA Mismatch Repair / genetics[mesh] OR CHEK2 protein, human[mesh] OR Chek2[tiab] OR

"Genetic Testing"[Mesh] OR “genetic test*” OR "Genetic Carrier Screening"[Mesh] OR “genetic carrier screen*” OR “genetic screen*” OR "Genetic Services"[Mesh] OR “Genetic service*” OR "Genetics, Medical"[Mesh] OR Genetic Variation[mesh] OR “Genetic variation*” OR Genetic Predisposition to Disease[mesh] OR “genetic predisposition to disease” OR DNA Mismatch Repair/genetics[mesh] OR “dna mismatch repair” OR Ethnic Groups / genetics[mesh])

### Scopus

Searched April 7, 2021

648 results

TITLE-ABS-KEY (brca OR brca1 OR brca2 OR "germline mutation*" OR "germ-line mutation*" OR TP53TG1 OR TP53 OR TP53TG5 OR p53 OR "rad52" OR "gene mutat*" OR "nucleotide sequenc*" OR "dna sequence analysis" OR "nanopore sequenc*" OR "Nucleotide sequen*" OR "dna repair" OR "li-fraumeni" OR "neoplasm dna" OR "Chek2" OR "genetic test*" OR "genetic carrier screen*" OR "genetic screen*" OR "Genetic service*" OR "medical genetics" OR "Genetic variation*" OR "genetic predisposition to disease" OR "dna mismatch repair") AND ("African descent" OR "African ancestry" OR "African Europeans*" OR "African Asians*" OR "African Americans*" OR "African migrants" OR "Southern African Development Community" OR "SADC" OR "African Caribbeans*" OR "African continental ancestry" OR "Africa South of the Sahara" OR "sub-sahara*" OR "subsahara*" OR "Angola" OR "Luanda" OR "Luanda" OR "Benin" OR "Porto-Novo" OR "Botswana" OR "Gaborone" OR "Burkina Faso" OR "Ouagadougou" OR "Burundi" OR "Gitega" OR "Cameroon" OR "Yaounde" OR "Central African Republic" OR "Bangui" OR "Chad" OR "N'Djamena" OR "Comoros" OR "Moroni" OR "Democratic Republic of the Congo" OR "Kinshasa" OR "Republic of the Congo" OR "Brazzaville" OR "Cote d'Ivoire" OR "Yamoussoukro" OR "Djibouti" OR "Equatorial Guinea" OR "Malabo" OR "Eritrea" OR "Asmara" OR "Eswatini" OR "Mbabane" OR "Ethiopia" OR "Addis Ababa" OR "Gabon" OR "Libreville" OR "Gambia" OR "Banjul" OR "Ghana" OR "Accra" OR "Guinea" OR "Conakry" OR "Guinea-Bissau" OR "Bissau" OR "Kenya" OR "Nairobi" OR "Lesotho" OR "Maseru" OR "Liberia" OR "Monrovia" OR "Madagascar" OR "Antananarivo" OR "Malawi" OR "Lilongwe" OR "Mali" OR "Bamako" OR "Mauritania" OR "Nouakchott" OR "Mauritius" OR "Port Louis" OR "Mozambique" OR "Maputo" OR "Namibia" OR "Windhoek" OR "Niger" OR "Niamey" OR "Nigeria" OR "Abuja" OR "Rwanda" OR "Kigali" OR "Sao Tome and Principe" OR "Sao Tome" OR "Senegal" OR "Dakar" OR "Seychelles" OR "Sierra Leone" OR "Freetown" OR "Somalia" OR "Mogadishu" OR "South Africa" OR "Pretoria" OR "South Sudan" OR "Juba" OR "Sudan" OR "Khartoum" OR "Tanzania" OR "Dodoma" OR "Togo" OR "Lomé" OR "Uganda" OR "Kampala" OR "Zambia" OR "Lusaka" OR "Zimbabwe" OR "Harare" OR "west-africa*" OR "west africa*" OR

"south african*" OR "east-africa*" OR "east africa*") AND ("breast neoplasm*" OR "breast cancer*" OR "breast neoplasm*" OR "cancer of the breast" OR "neoplasm of the breast" OR "neoplasms of the breast" OR "ductal breast carcinoma*" OR "mammary cancer*" OR "mammary neoplasm*" OR "breast malignant neoplasm*" OR "breast malignant tumor*" OR "breast malignant tumour*" OR "breast tumor*" OR "breast tumour*")

### CINAHL

Searched April 7, 2021

137 results

((MH "Africa, Southern+") OR (MH "Africa, Western+") OR (MH "Africa South of the Sahara+") OR (MH "Africa, Eastern") OR (MH "Africa, Central+") OR ("African descent" OR "African ancestry" OR "African Europeans*" OR "African Asians*" OR "African Americans*" OR "African migrants" OR "Southern African Development Community" OR "SADC" OR "African Caribbeans*" OR african continental ancestry group[mesh] OR “African continental ancestry” OR "Africa South of the Sahara"[Mesh] OR sub-sahara* OR subsahara* OR Angola OR Luanda OR Luanda OR Benin OR Porto-Novo OR Botswana OR Gaborone OR Burkina Faso OR Ouagadougou OR Burundi OR Gitega OR Cameroon OR Yaounde OR "Central African Republic" OR Bangui OR Chad OR N'Djamena OR Comoros OR Moroni OR "Democratic Republic of the Congo" OR Kinshasa OR "Republic of the Congo" OR Brazzaville OR "Cote d'Ivoire" OR Yamoussoukro OR Djibouti OR "Equatorial Guinea" OR Malabo OR Eritrea OR Asmara OR Eswatini OR Mbabane OR Ethiopia OR "Addis Ababa" OR Gabon OR Libreville OR Gambia OR Banjul OR Ghana OR Accra OR Guinea OR Conakry OR Guinea- Bissau OR Bissau OR Kenya OR Nairobi OR Lesotho OR Maseru OR Liberia OR Monrovia OR Madagascar OR Antananarivo OR Malawi OR Lilongwe OR Mali OR Bamako OR Mauritania OR Nouakchott OR Mauritius OR Port Louis OR Mozambique OR Maputo OR Namibia OR Windhoek OR Niger OR Niamey OR Nigeria OR Abuja OR Rwanda OR Kigali OR "Sao Tome and Principe" OR "Sao Tome" OR Senegal OR Dakar OR Seychelles OR "Sierra Leone" OR Freetown OR Somalia OR Mogadishu OR "South Africa" OR Pretoria OR South Sudan OR Juba OR Sudan OR Khartoum OR Tanzania OR Dodoma OR Togo OR Lomé OR Uganda OR Kampala OR Zambia OR Lusaka OR Zimbabwe OR Harare OR "west-africa*" OR "west africa*" OR "south african*" OR "east-africa*" OR "east africa*") AND (brca OR brca1 OR brca2 OR "germline mutation*" OR "germ-line mutation*" OR TP53TG1 OR TP53 OR TP53TG5 OR p53 OR "rad52" OR "gene mutat*" OR "nucleotide sequenc*" OR "dna sequence analysis" OR "nanopore sequenc*" OR "Nucleotide sequen*" OR "dna repair" OR "li-fraumeni" OR "neoplasm dna" OR "Chek2" OR "genetic test*" OR "genetic carrier screen*" OR "genetic screen*" OR "Genetic service*" OR "medical genetics" OR genetic* OR "Genetic variation*" OR "genetic predisposition to disease" OR "dna mismatch repair" OR (MH "Rapid Sequence Induction and Intubation") OR (MH "Sequence Analysis+") OR (MH "Genes, BRCA") OR (MH "Genetic Screening") OR (MH "Genetic Techniques+") OR (MH "Genetic Research+") OR (MH "Genetic Variation+") OR (MH "Genetic Markers")) AND ("breast neoplasm*" OR "breast cancer*" OR "breast neoplasm*" OR "cancer of the breast" OR "neoplasm of the breast" OR "neoplasms of the breast" OR "ductal breast carcinoma*" OR "mammary cancer*" OR "mammary neoplasm*" OR "breast malignant neoplasm*" OR "breast malignant tumor*" OR "breast malignant tumour*" OR "breast tumor*" OR "breast tumour*") OR (MH "Breast Neoplasms+") )

### Cochrane Database

Searched April 7, 2021

47 results

(brca OR brca1 OR brca2 OR "germline mutation*" OR "germ-line mutation*" OR TP53TG1 OR TP53 OR TP53TG5 OR p53 OR "rad52" OR "gene mutat*" OR "nucleotide sequenc*" OR "dna sequence analysis" OR "nanopore sequenc*" OR "Nucleotide sequen*" OR "dna repair" OR "li-fraumeni" OR "neoplasm dna" OR "Chek2" OR "genetic test*" OR "genetic carrier screen*" OR "genetic screen*" OR "Genetic service*" OR "medical genetics" OR "Genetic variation*" OR "genetic predisposition to disease" OR "dna mismatch repair") AND ("African descent" OR "African ancestry" OR "African Europeans*" OR "African Asians*" OR "African Americans*" OR "African migrants" OR "Southern African Development Community" OR "SADC" OR "African Caribbeans*" OR "African continental ancestry" OR "Africa South of the Sahara" OR "sub-sahara*" OR "subsahara*" OR "Angola" OR "Luanda" OR "Luanda" OR "Benin" OR "Porto-Novo" OR "Botswana" OR "Gaborone" OR "Burkina Faso" OR "Ouagadougou" OR "Burundi" OR "Gitega" OR "Cameroon" OR "Yaounde" OR "Central African Republic" OR "Bangui" OR "Chad" OR "N'Djamena" OR "Comoros" OR "Moroni" OR "Democratic Republic of the Congo" OR "Kinshasa" OR "Republic of the Congo" OR "Brazzaville" OR "Cote d'Ivoire" OR "Yamoussoukro" OR "Djibouti" OR "Equatorial Guinea" OR "Malabo" OR "Eritrea" OR "Asmara" OR "Eswatini" OR "Mbabane" OR "Ethiopia" OR "Addis Ababa" OR "Gabon" OR "Libreville" OR "Gambia" OR "Banjul" OR "Ghana" OR "Accra" OR "Guinea" OR "Conakry" OR "Guinea-Bissau" OR "Bissau" OR "Kenya" OR "Nairobi" OR "Lesotho" OR "Maseru" OR "Liberia" OR "Monrovia" OR "Madagascar" OR "Antananarivo" OR "Malawi" OR "Lilongwe" OR "Mali" OR "Bamako" OR "Mauritania" OR "Nouakchott" OR "Mauritius" OR "Port Louis" OR "Mozambique" OR "Maputo" OR "Namibia" OR "Windhoek" OR "Niger" OR "Niamey" OR "Nigeria" OR "Abuja" OR "Rwanda" OR "Kigali" OR "Sao Tome and Principe" OR "Sao Tome" OR "Senegal" OR "Dakar" OR "Seychelles" OR "Sierra Leone" OR "Freetown" OR "Somalia" OR "Mogadishu" OR "South Africa" OR "Pretoria" OR "South Sudan" OR "Juba" OR "Sudan" OR "Khartoum" OR "Tanzania" OR "Dodoma" OR "Togo" OR "Lomé" OR "Uganda" OR "Kampala" OR "Zambia" OR "Lusaka" OR "Zimbabwe" OR "Harare" OR "west-africa*" OR "west africa*" OR "south african*" OR "east-africa*" OR "east africa*") AND ("breast neoplasm*" OR "breast cancer*" OR "breast neoplasm*" OR "cancer of the breast" OR "neoplasm of the breast" OR "neoplasms of the breast" OR "ductal breast carcinoma*" OR "mammary cancer*" OR "mammary neoplasm*" OR "breast malignant neoplasm*" OR "breast malignant tumor*" OR "breast malignant tumour*" OR "breast tumor*" OR "breast tumour*")

### EMBASE

Searched April 7, 2021

1,002 results

(brca OR brca1 OR brca2 OR 'germline mutation*' OR 'germ-line mutation*' OR tp53tg1 OR tp53 OR tp53tg5 OR p53 OR 'rad52' OR 'gene mutat*' OR 'nucleotide sequenc*' OR 'dna sequence analysis' OR 'nanopore sequenc*' OR 'nucleotide sequen*' OR 'dna repair' OR 'li- fraumeni' OR 'neoplasm dna' OR 'chek2' OR 'genetic test*' OR 'genetic carrier screen*' OR 'genetic screen*' OR 'genetic service*' OR 'medical genetic*' OR 'genetic variation*' OR 'genetic predisposition to disease' OR 'dna mismatch repair' OR 'brca1 protein'/exp OR 'brca2 protein'/exp OR 'genetic screening'/exp OR 'protein p53'/exp OR 'gene sequence'/exp OR 'nanopore sequencing'/exp OR 'li-fraumeni syndrome'/exp OR 'dna repair'/exp OR 'heterozygote detection'/exp OR 'genetic predisposition'/exp) AND ('breast tumor'/exp OR 'breast cancer*' OR 'breast neoplasm*' OR 'cancer of the breast' OR 'neoplasm of the breast' OR 'neoplasms of the breast' OR 'ductal breast carcinoma*' OR 'mammary cancer*' OR 'mammary neoplasm*' OR 'breast malignant neoplasm*' OR 'breast malignant tumor*' OR 'breast malignant tumour*' OR 'breast tumor*' OR 'breast tumour*') AND ('african descent' OR 'african ancestry' OR 'african europeans*' OR 'african asians*' OR 'african americans*' OR 'african migrants' OR 'southern african development community' OR 'sadc' OR 'african caribbeans*' OR 'african continental ancestry' OR 'africa south of the sahara' OR 'sub-sahara*' OR 'subsahara*' OR 'angola'/exp OR 'angola' OR 'luanda' OR 'benin'/exp OR 'benin' OR 'porto-novo' OR 'botswana'/exp OR 'botswana' OR 'gaborone' OR 'burkina faso'/exp OR 'burkina faso' OR 'ouagadougou' OR 'burundi'/exp OR 'burundi' OR 'gitega' OR 'cameroon'/exp OR 'cameroon' OR 'yaounde' OR 'central african republic'/exp OR 'central african republic' OR 'bangui' OR 'chad'/exp OR 'chad' OR 'n djamena' OR 'comoros'/exp OR 'comoros' OR 'moroni' OR 'democratic republic of the congo'/exp OR 'democratic republic of the congo' OR 'kinshasa' OR 'republic of the congo' OR 'brazzaville' OR 'cote d ivoire'/exp OR 'cote d ivoire' OR 'yamoussoukro' OR 'djibouti'/exp OR 'djibouti' OR 'equatorial guinea'/exp OR 'equatorial guinea' OR 'malabo' OR 'eritrea'/exp OR 'eritrea' OR 'asmara' OR 'eswatini'/exp OR 'eswatini' OR 'mbabane' OR 'ethiopia'/exp OR 'ethiopia' OR 'addis ababa' OR 'gabon'/exp OR 'gabon' OR 'libreville' OR 'gambia'/exp OR 'gambia' OR 'banjul' OR 'ghana'/exp OR 'ghana' OR 'accra' OR 'guinea'/exp OR 'guinea' OR 'conakry' OR 'guinea-bissau'/exp OR 'guinea-bissau' OR 'bissau' OR 'kenya'/exp OR 'kenya' OR 'nairobi' OR 'lesotho'/exp OR 'lesotho' OR 'maseru' OR 'liberia'/exp OR 'liberia' OR 'monrovia' OR 'madagascar'/exp OR 'madagascar' OR 'antananarivo' OR 'malawi'/exp OR 'malawi' OR 'lilongwe' OR 'mali'/exp OR 'mali' OR 'bamako' OR 'mauritania'/exp OR 'mauritania' OR 'nouakchott' OR 'mauritius'/exp OR 'mauritius' OR 'port louis' OR 'mozambique'/exp OR 'mozambique' OR 'maputo' OR 'namibia'/exp OR 'namibia' OR 'windhoek' OR 'niger'/exp OR 'niger' OR 'niamey' OR 'nigeria'/exp OR 'nigeria' OR 'abuja' OR 'rwanda'/exp OR 'rwanda' OR 'kigali' OR 'sao tome and principe'/exp OR 'sao tome and principe' OR 'sao tome' OR 'senegal'/exp OR 'senegal' OR 'dakar' OR 'seychelles'/exp OR 'seychelles' OR 'sierra leone'/exp OR 'sierra leone' OR 'freetown' OR 'somalia'/exp OR 'somalia' OR 'mogadishu' OR 'south africa'/exp OR 'south africa' OR 'pretoria' OR 'south sudan'/exp OR 'south sudan' OR 'juba' OR 'sudan'/exp OR 'sudan' OR 'khartoum' OR 'tanzania'/exp OR 'tanzania' OR 'dodoma' OR 'togo'/exp OR 'togo' OR 'lomé' OR 'uganda'/exp OR 'uganda' OR 'kampala' OR 'zambia'/exp OR 'zambia' OR 'lusaka' OR 'zimbabwe'/exp OR 'zimbabwe' OR 'harare' OR 'west-africa*' OR 'west africa*' OR 'south african*' OR 'east-africa*' OR 'east africa*' OR 'africa south of the sahara'/exp)

## APPENDIX 2. SCOPING REVIEW DATA EXTRACTION FORM

1. Manuscript title
2. Country of institutional affiliation for first author:
United States of America (187) … Zimbabwe (1357)
3. Record first-author institutional affiliation:
4. Country of institutional affiliation for last author:
United States of America (187) … Zimbabwe (1357)
5. Record last-author institutional affiliation:
6. Where was the research conducted?
7. Record study type:
  a. Literature review (meta-analysis, systematic, scoping)
  b. Randomized controlled trial (RCT)
  c. Retrospective analysis (cross-sectional, cohort)
  d. Prospective (cross-sectional, cohort)
  e. Mixed methods
  f. Qualitative study
  g. Other
8. Was informed consent obtained in this study?
  a. Yes
  b. No
  c. Unsure
9. Please elaborate on the informed consent process:
10. What germline mutation was studied?
  a. None
  b. BRCA1
  c. BRCA2
  d. TP53
  e. RADS1
  f. CDH1
  g. NDN
  h. CHEK2
  i. FANCA
  j. MRE11A
  k. SPINK1
  l. MLH1
  m. ATM
  n. Other (specify)
11. Was germline genetic testing conducted as part of this study?
  a. Yes
  b. No
12. What samples were used for genetic testing, if conducted?
  a. Blood
  b. Saliva
  c. Unknown
  d. Other (specify)
13. How were germline mutations tested?
  a. SNPs
  b. PCR
  c. Next-generation sequencing
  d. Sanger sequencing
  e. Unsure
  f. Other (specify)
14. Record specific testing platform or sequencing model if applicable:
15. Did they use an existing database?
  a. No
  b. Yes
  c. Unsure
16. Record database
  a. SEER
  b. Cancer Genome Atlas
  c. Breast Cancer Association Consortium
  d. Consortium of Investigators of Modifiers of BRCA1/2 (CIMBA)
  e. Women's Circle of Health Study
  f. Breast Cancer Family Registry and the University of Chicago
  g. Other State Cancer Registry (indicate state)
  h. Other (specify)
17. Which racial/ethnic categories were used?
  a. African American (AA)
  b. African ancestry
  c. Black African
  d. African or African descent
  e. Black American
  f. Non-Hispanic Black
  g. Black
  h. Other (specify)
18. Was “race,” “ethnicity,” or other element describing these variable defined in the article?
  a. Yes
  b. No
  c. Unsure
19. How was it defined?
20. Was “racism” ever mentioned in the manuscript
  a. Yes
  b. No
21. How was “racism” mentioned? Copy and paste the sentence or paragraph for context.
22. Record population size (No.):
  Total study population:
  African American population:
  White population:
  Other race/ethnicity or subpopulation:
  Other race/ethnicity or subpopulation:
  Other race/ethnicity or subpopulation:
  Other race/ethnicity or subpopulation:
  Other race/ethnicity or subpopulation:
23. Was genetic counseling implemented or evaluated?
  a. Yes
  b. No
24. Did this study evaluate perspectives on genetic testing?
  a. Yes
  b. No
25. Method for evaluation:
  a. Interviews
  b. Phone survey
  c. Survey
  d. Focus groups
  e. Electronic medical record
  f. Other (specify)
26. What were the outcomes/findings of the paper?
27. Was there a bioethical component to the manuscript?
  a. Yes
  b. Maybe
  c. No
28. What ethical elements were raised?
  a. Equity
  b. Autonomy
  c. Cost
  d. Data ownership
  e. Hesitance
  f. Unintentional harm
  g. Social or cultural norms
  h. Religion
  i. Other
29. Any additional comments about the manuscript:
